# Cardiovascular Effects of a Glycosylated Flavonoids-Rich Leaf Extract from Brazilian *Erythroxylum campestre*: A Potential Health Bio-Input

**DOI:** 10.3390/ph17111456

**Published:** 2024-10-30

**Authors:** Letícia Henrique Dantas Gomes de Lima, Marcos Willian Francelino Gomes, Thays Siqueira de Sá Curado, Lara Marques Naves, Stefanne Madalena Marques, Marilene Silva Oliveira, John Ihayi Ogbu, Antonio Carlos Severo Menezes, Giuliana Muniz Vila Verde, James Oluwagbamigbe Fajemiroye, Gustavo Rodrigues Pedrino

**Affiliations:** 1 Department of Physiological Science, Biological Sciences Institute, Federal University of Goiás, Estrada do Campus, s/n, Goiânia 74690-900, GO, Brazil; leticiahenrique@discente.ufg.br (L.H.D.G.d.L.); laramarques.naves@gmail.com (L.M.N.); stefannemadalena@gmail.com (S.M.M.); 2 Natural Products Laboratory, State University of Goiás, BR-153 3105 Fazenda Barreiro do Meio, Anápolis 75132-903, GO, Brazil; marcoswillian_fg@hotmail.com (M.W.F.G.); antonio.menezes@ueg.br (A.C.S.M.); 3 Bioproducts and Synthesis Research Laboratory, State University of Goiás, BR-153 3105 Fazenda Barreiro do Meio, Anápolis 75132-903, GO, Brazil; thays_piri@hotmail.com (T.S.d.S.C.); marilenes36@gmail.com (M.S.O.); giuliana.muniz@ueg.br (G.M.V.V.); 4 Laboratory of Pharmacology of Natural and Synthetic Products, Institute of Biological Sciences, Federal University of Goiás, Goiânia 74690-900, GO, Brazil; ogbu11john@gmail.com (J.I.O.); jamesfajemiroye@ufg.br (J.O.F.)

**Keywords:** bioactivity, hypertension, medicinal plants, phytochemical, vascular resistance

## Abstract

**Background**: Bioactivity assessments of plant-derived products can benefit human and animal health, especially in regions with vast plant diversity. This study focused on chemical and cardiovascular analyses of *Erythroxylum campestre* A. St. Hil. leaf extracts. **Methods**: High-performance liquid chromatography, liquid chromatography coupled with mass spectrometry, and nuclear magnetic resonance spectroscopy were used to elucidate the structures of the flavonoids in *E. campestre*. The *E. campestre* methanolic fraction (ECM-ppt-M; at doses of 1, 2, 3, and 6 mg∙kg^−1^ or vehicle) was administered intravenously to normotensive and spontaneously hypertensive rats (SHRs), and we recorded the mean arterial pressure (MAP), heart rate (HR), renal vascular resistance (RVR), and aortic vascular resistance (AVC). **Results**: The ECM-ppt-M extract demonstrated significant antihypertensive activity, as evidenced by reductions in MAP, RVR, and AVR, with effects that were particularly pronounced in SHRs. Following the establishment of these cardiovascular effects, phytochemical analysis revealed the presence of glycosylated flavonoids, which are likely contributors to the observed antihypertensive properties of the extract. **Conclusions**: The notable reductions in MAP and vascular resistance observed with ECM-ppt-M treatment suggest its antihypertensive effect. These findings demonstrate the potential therapeutic value of this extract with regard to the treatment of hypertension. Future studies on ECM may provide a promising therapeutic alternative capable of reducing the risk of toxicity and adverse effects associated with synthetic drugs.

## 1. Introduction

Although the subject of plant-derived products has been widely explored, the collection of plant materials, extraction, phytochemical, and pharmacological characterization always involves a lot of effort [1,2,3]. The use of plants for medicinal purposes has been a long-standing practice across diverse population groups [1]. Information about the medicinal uses of different plant classes and their applications constitutes empirical knowledge, passed down from generation to generation [2]. The importance of medicinal plants as therapeutic resources has grown considerably, especially in light of the limitations of synthetic drugs [4,5,6] The World Health Organization estimates that medication errors result in an annual cost of $42 billion, highlighting both the financial and health-related risks associated with conventional treatments [7]. Moreover, synthetic drugs often come with significant toxicological concerns and adverse side effects [3,8,9]. Consequently, natural therapies are gaining recognition as viable alternatives that may offer safer, more holistic approaches to health management.

Brazil is among the world’s most biodiverse places in terms of plants, with over 34,916 documented species, 55% of which are endemic [10]. It is estimated that only 8% of the plant species native to the Brazilian flora have been studied for their bioactive compounds, and 1100 plant species have been evaluated for their medicinal properties [11]. Erythroxylaceae is prominent among the diverse families of plant species in Brazil that produce important secondary metabolites [12]. This family comprises four distinct genera, which are distributed in tropical and subtropical regions worldwide. The genus *Erythroxylum* comprises approximately 250 species [13].

Despite the widespread use of Erythroxylaceae, the diversity within the Erythroxylaceae family remains poorly studied [14]. Several species of the Erythroxylum genus are recognized for their medicinal properties, primarily due to the presence metabolites in their leaves [15]. Traditionally, Erythroxylum has been used as an antidiarrheal, an antipyretic, a diuretic, an asthma inhalant, and a vermifuge [16,17]. Additionally, various species within the genus have been utilized to treat conditions such as amenorrhea, digestive and kidney disorders, flu, hemorrhage, sinusitis, and fatigue [18,19]. Notably, compounds like tropane alkaloids and phenolic constituents, including rutin, tannins, and flavonoids, have been identified within this genus, underscoring its broad therapeutic potential [20].

While phenolic compounds are known for their applications in the treatment of cardiovascular diseases, the specific anti-hypertensive effects of *E. campestre* remain unexplored. Given the potential of flavonoids in terms of bioactivity, this research aims to elucidate the effects of *E. campestre* on hypertensive rat models, contributing to the broader field of plant-based therapies for cardiovascular conditions. By focusing on this ethnobotanically significant species, this study explores its potential as a natural, sustainable alternative for cardiovascular disease treatment.

## 2. Results

### 2.1. Fractionation of the Crude Extract

After processing *E. campestre* methanolic extract (ECM) samples, fractionation was performed. The masses obtained after fractionation and their yields are listed in Table 1 and Table 2, respectively. When analyzing the yields of the precipitated material fractions, the fraction in which methanol was used as the solvent (ECM-ppt-M) obtained the highest yield, as shown in Table 2.

### 2.2. The Evaluation of Cardiovascular Changes Induced by the Infusion of the Methanolic Fraction of E. campestre

**Figure 1:**
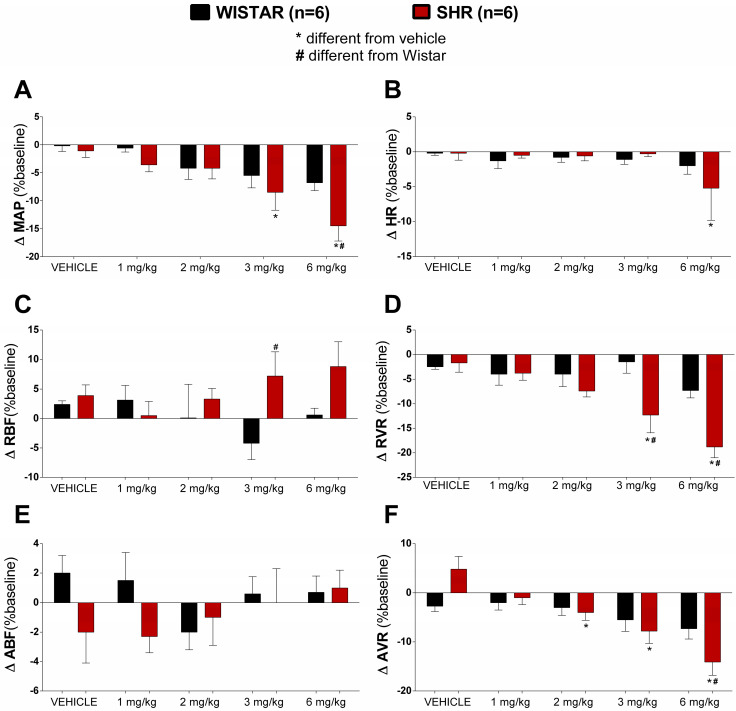
The analysis of cardiovascular effects. Changes in variations in mean arterial pressure (MAP, % baseline; (**A**)), heart rate (HR, %baseline; (**B**)), renal blood flow (RBF, % baseline; (**C**)), renal vascular resistance (RVR, % baseline; (**D**)), aortic blood flow (ABF % baseline; (**E**)), and aortic vascular resistance (AVR, % baseline; (**F**)) in Wistar (*n* = 6) and SHR (*n* = 6) groups that received a bolus infusion of varying doses of ECM-ppt-M obtained from the fractionation of precipitated material of *E. campestre* methanolic extract precipitate (ECM-ppt-M). * different from vehicle. ^#^ different from Wistar. *p* < 0.05.

The vehicle (5% DMSO in distilled water), which served as a control, and four different doses (1, 2, 3, and 6 mg∙kg^−1^) of *E. campestre* methanolic extract precipitate (ECM-ppt-M) were administered. The cardiovascular parameters recorded included mean arterial pressure (MAP), heart rate (HR), renal blood flow (RBF), renal vascular resistance (RVR), aortic blood flow (ABF), and aortic vascular resistance (AVR). In normotensive animals, no significant differences were observed between the ECM-ppt-M-treated animals and the vehicle-treated group in MAP (Δ vehicle: −0.2 ± 1.0; Δ 1 mg/kg: −0.6 ± 0.7; Δ 2 mg/kg: −4.2 ± 2.0; Δ 3 mg/kg: −5.5 ± 2.2; Δ 6 mg/kg: −6.8 ± 1.4% relative to baseline; Figure 1A), HR (Δ vehicle: −0.2 ± 0.3; Δ 1 mg/kg: −1.3 ± 1.1; Δ 2 mg/kg: −0.8 ± 0.7; Δ 3 mg/kg: −1.1 ± 0.7; Δ 6 mg/kg: −2.0 ± 1.2% relative to baseline; Figure 1B), RBF (Δ vehicle: 2.4 ± 0.6; Δ 1 mg/kg: 3.1 ± 2.5; Δ 2 mg/kg: 0.1 ± 5.7; Δ 3 mg/kg: −4.2 ± 2.8; Δ 6 mg/kg: 0.6 ± 1.1% relative to baseline; Figure 1C), RVR (Δ vehicle: −2.5 ± 0.5; Δ 1 mg/kg: −4.0 ± 2.2; Δ 2 mg/kg: −4.0 ± 2.5; Δ 3 mg/kg: −1.5 ± 2.3; Δ 6 mg/kg: −7.3 ± 1.5% relative to baseline; Figure 1D), ABF (Δ vehicle: 2.0 ± 1.2; Δ 1 mg/kg: 1.5 ± 1.9; Δ 2 mg/kg: −2.0 ± 1.2; Δ 3 mg/kg: 0.6 ± 1.1; Δ 6 mg/kg: 0.7 ± 1.1% relative to baseline; Figure 1E), and AVR (Δ vehicle: −2.7 ± 1.1; Δ 1 mg/kg: −2.0 ± 1.5; Δ 2 mg/kg: −3.0 ± 1.6; Δ 3 mg/kg: −5.5 ± 2.3; Δ 6 mg/kg: −7.3 ± 2.1% relative to baseline; Figure 1F).

In the spontaneously hypertensive rat (SHR) group, ECM-ppt-M treatment reduced MAP (Δ vehicle: −1.1 ± 1.1 vs. −8.5 ± 3.2 and −14.5 ± 2.7% relative to baseline; after doses of 3 mg/kg and 6 mg/kg of ECM-ppt-M, respectively; *p <* 0.05; Figure 1A), RVR (Δ vehicle: −1.7 ± 1.9 vs. −12.3 ± 3.6 and −18.8 ± 2.2% relative to baseline; after doses of 3 mg/kg and 6 mg/kg, respectively; *p <* 0.05; Figure 1D), and AVR (Δ vehicle: 4.8 ± 2.6 vs. −4.0 ± 1.6; −7.8 ± 2.5, and −14.1 ± 2.7% relative to baseline; after doses of 2 mg/kg, 3 mg/kg, and 6 mg/kg, respectively; *p <* 0.05; Figure 1F). Only the highest dose of the extract elicited bradycardia (Δ vehicle: −0.2 ± 1.0 vs. −5.2 ± 4.6% relative to baseline; after doses of 6 mg/kg, *p <* 0.05; Figure 1B). No significant differences were found between the groups in RBF (Δ vehicle: 3.9 ± 1.8; Δ 1 mg/kg: 0.5 ± 2.4; Δ 2 mg/kg: 3.3 ± 1.8; Δ 3 mg/kg: 7.2 ± 4.1; Δ 6 mg/kg: 8.8 ± 4.2% relative to baseline; Figure 1C) and ABF (Δ vehicle: −2.0 ± 2.1; Δ 1 mg/kg: −2.3 ± 1.1; Δ 2 mg/kg: −1.0 ± 1.9; Δ 3 mg/kg: 0.0 ± 2.3; Δ 6 mg/kg: 1.0 ± 1.2% relative to baseline; Figure 1E).

Comparing the response between hypertensive and normotensive animals, we observed that the ECM-ppt-M treatment elicited significant changes in MAP (Δ Wistar: −6.8 ± 1.4 vs. Δ SHRs: −14.5 ± 2.7% relative to baseline; after the dose of 6 mg/kg; *p <* 0.05; Figure 1A), RBF (Δ Wistar: −4.2 ± 2.8 vs. Δ SHRs: 7.2 ± 4.1% relative to baseline; after the dose of 3 mg/kg; *p <* 0.05; Figure 1C), RVR (Δ Wistar: −1.5 ± 2.3 and −7.3 ± 1.5% relative to baseline vs. Δ SHRs: −12.3 ± 3.6 and −18.8 ± 2.2% relative to baseline; after doses of 3 mg/kg and 6 mg/kg, respectively; *p <* 0.05; Figure 1D) and AVR (Δ Wistar: −7.3 ± 2.1% relative to baseline vs. Δ SHRs: −14.1 ± 2% relative to baseline; after the dose of 6 mg/kg; *p <* 0.05; Figure 1F). HR and ABF responses did not differ between the groups.

### 2.3. Analysis of High-Performance Liquid Chromatography—HPLC/UV

**Figure 2:**
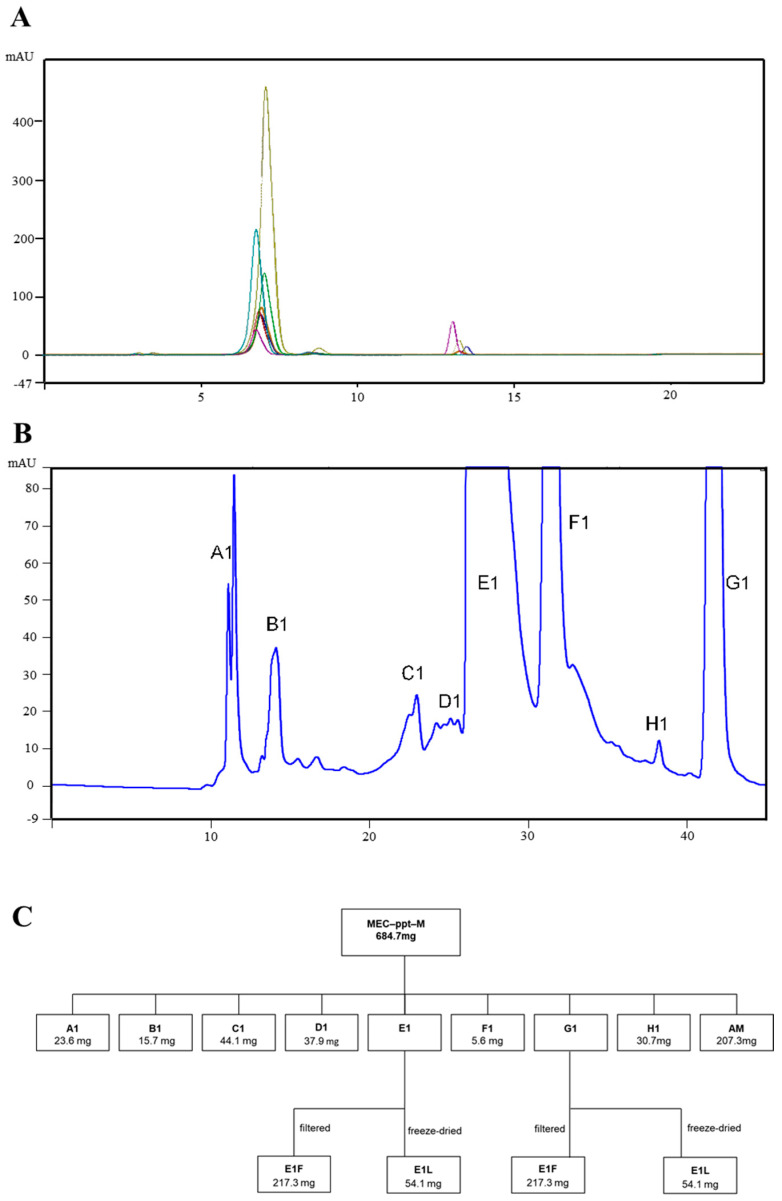
Analysis using high-performance liquid chromatography (HPLC/UV)/UV. Chromatograms obtained for samples VI, VII, VIII, IX, X, XI, and XIII: (**A**) the enlargement of the chromatogram for the isolation of constituents from ECM-ppt-M; (**B**) the identification of the collected compounds; and (**C**) a flowchart of the isolation of ECM-M by HPLC/UV.

The chromatographic analysis of the ECM-ppt-M fraction proved to be quite efficient in terms of isolating the constituents, taking no more than 45 min for each analysis. The analysis of the eight samples showed peaks similar to those of the previous analysis. Therefore, we decided to use ECM-ppt-M (approximately 25 mg·mL^−1^). The eight fractions were named A1, B1, C1, D1, E1, F1, G1, and H1, as shown in the chromatograms in Figure 2A,B.

After removing the solvent from the isolated substances and performing low-pressure rotary evaporation for methanol removal, precipitation was observed in the fifth (E1) and seventh (G1) fractions, which were then filtered and stored (Figure 2C).

### 2.4. Structural Elucidation

#### 2.4.1. Structural Analysis by Mass Spectrometry

**Figure 3:**
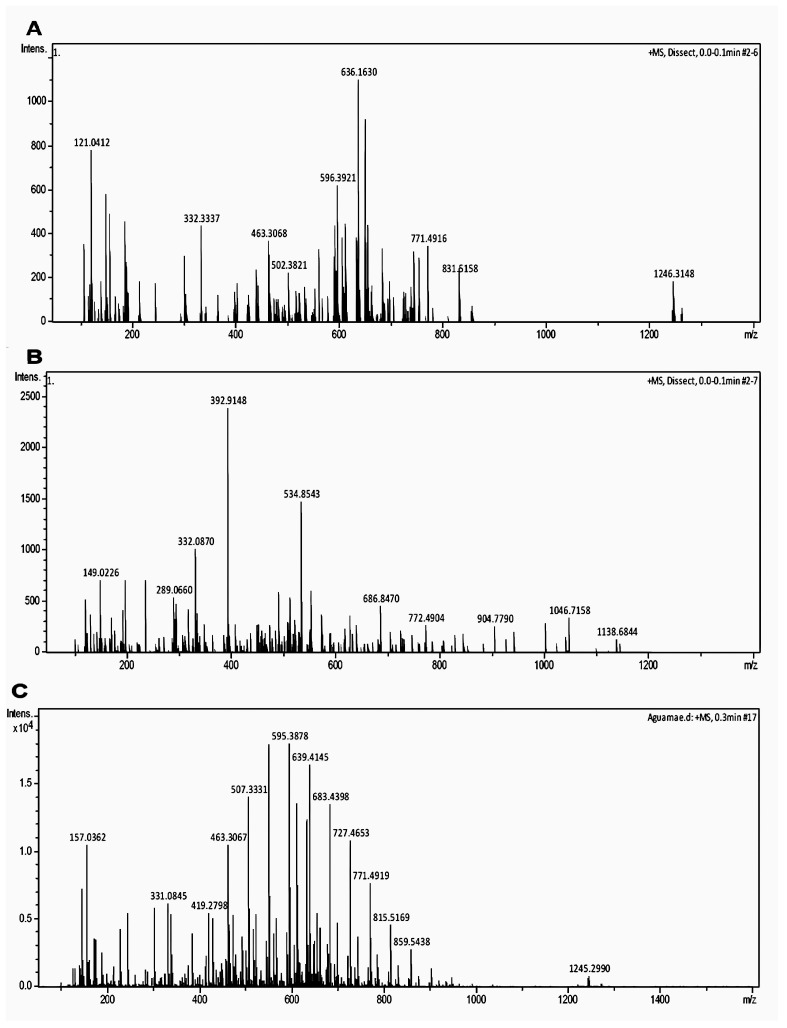
The characterization of the negative-ion electrospray ionization mass spectra of fractions E1F (**A**), F1 (**B**), and AM (**C**). The x-axis shows the *m*/*z* and y-axis shows relative abundance (100%), which appears as intensity.

To elucidate which substances were present in the fractions, mass spectrometry, hydrogen, and carbon nuclear magnetic resonance were employed for the E1F, F1, and AM fractions (Figure 3A–C). The results of this analysis are summarized in Table 3. Based on the literature, the base peaks of the E1F, F1, and AM samples (634.1579, 595.3916, 595.3815, and 633.1480 *m*/*z*, respectively) were similar to those of rutin and kaempferol (610.518 and 594.518, respectively).

#### 2.4.2. Structural Analysis by Hydrogen and Carbon Nuclear Magnetic Resonance

To investigate the presence of glycosylated flavonoids in the ECM-ppt-M fraction, nuclear magnetic resonance (NMR) analysis was performed on samples E1F, F1, and AM.

**Figure 4:**
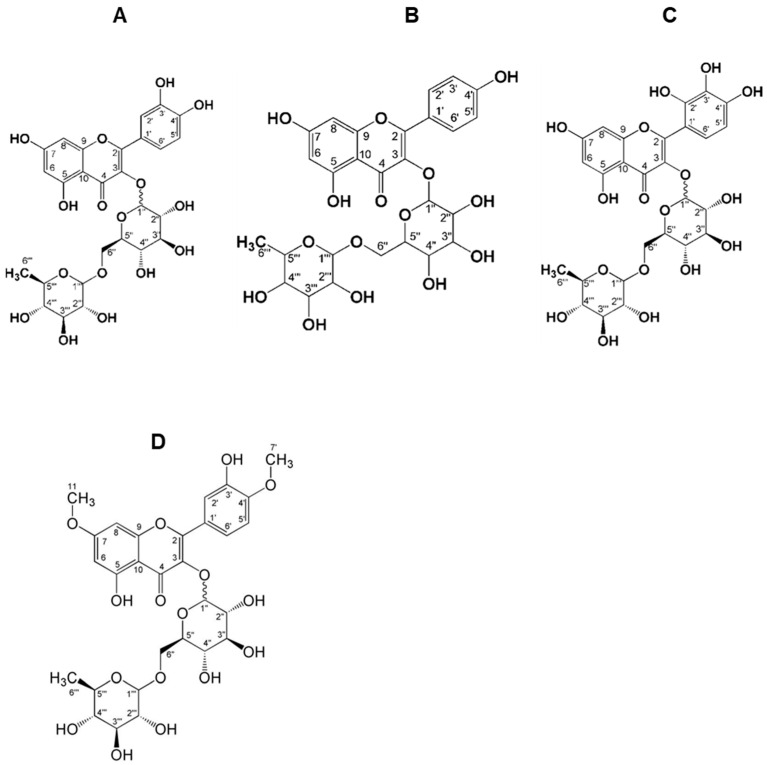
The characterization of chemical structures of compounds from *E. campestre*. The chemical structure of the flavonoid rutin (**A**), the chemical structure of the flavonoid kaempferol-3-rutinoside (**B**), the chemical structure of the novel flavonoid (**C**), and the chemical structure of the flavonoid ombuin-3-rutinoside (**D**).

The E1F fraction had a solid physical state, an amorphous structure, a yellow color, and a mass of 217.3 mg. The ^1^H NMR spectrum in the aromatic hydrogen region shows a doublet at δH 7.57 (*J =* 2.15 Hz), referring to hydrogen 2′; a double doublet at δH 7.53 (*J =* 8.61 Hz and 2.35 Hz), referring to hydrogen 6′; a doublet at δH 6.78 (*J =* 8.41 Hz), referring to hydrogen 5′; a doublet at δH 6.31 (*J =* 2.01 Hz), referring to hydrogen 8; and a doublet at δH 6.12 (*J =* 2.06 Hz), referring to hydrogen 6. This is evidently a characteristic model of quercetin derivatives. The ^1^H NMR spectrum also indicates the presence of two sugar units through anomeric hydrogen signals at δH 5.00 (*J =* 7.93 Hz) and at δH 4.42 (*J =* 1.21 Hz), attributed, respectively, to hydrogens 1″ and 1‴ of carbonyl hydrogens in the region of δH 3.15–3.71. The ^13^C NMR spectrum shows a signal at δC 178.0, which is typical of chelated carbonyl, as well as signals in the region of δC 67.1–76.8 and two signals at δC 103.28 and 101.02, referring to the anomeric carbons 1″ and 1‴. This confirms the existence of two sugar units in this flavonoid (Figure 4A).

The F1 fraction had a solid physical state, an amorphous structure, yellow color, and a mass of 5.6 mg. In the ^1^H NMR spectrum, the region of aromatic hydrogen shows a doublet at δ 6.43 (*J =* 6.25 Hz), referring to hydrogen 6; a doublet at δ 6.25 (*J =* 6.25 Hz), referring to hydrogen 8; a doublet at δ 8.08 (*J =* 8.64 Hz), referring to hydrogen 2′; a doublet at δ 6.90 (*J =* 8.09 Hz), referring to hydrogen 3′; a doublet at δ 6.93 (*J =* 7.35 Hz), referring to hydrogen 5′; and a doublet at δ 8.10 (*J =* 9.00 Hz), referring to hydrogen 6′. A characteristic model of kaempferol derivatives was presented. The ^1^H NMR spectrum also reveals the presence of two sugar units through anomeric hydrogen signals, δ 5.14 (*J =* 7.16 Hz) and δ 4.53 (*J =* 1.01 Hz). This is attributed to hydrogens 1″ and 1‴ of carbonyl hydrogens in the region of δ 3.06–3.60 (Figure 4B).

The AM fraction had a solid physical state, an amorphous structure, a yellow color, and a mass of 207.3 mg. In the ^1^H NMR spectrum (Table S1 and Figure S1), the integral of the peak intensities indicates the presence of more than one substance. This is coherent because the AM fraction is the mother liquor, where all parts that were not collected in the HPLC-RP were concentrated. In this study, two structures were proposed for the substances present in this fraction. We proposed that the minor compound was AM1 and the major compound was AM2. By analyzing the ^1^H NMR spectrum, it was also possible to deduce the presence of rutin in this fraction. AM1 is a novel compound. In the ^1^H NMR spectrum, the region of aromatic hydrogen shows a doublet at δ 6.35 (*J =* 2.13 Hz), referring to hydrogen 6; a doublet at δ 6.60 (*J =* 1.86 Hz), referring to hydrogen 8; a doublet at δ 7.05 (*J =* 8.73 Hz), referring to hydrogen 3′; a doublet at δ 8.08 (*J =* 9.07 Hz), referring to hydrogen 6′; and a doublet at δ 6.60 (*J =* 1.86 Hz). A characteristic model of quercetin derivatives is presented. The ^1^H NMR spectrum also exhibited the presence of two sugar units through anomeric hydrogen signals at δH 5.22 (*J =* 7.88 Hz) and δH 4.53 (*J =* 1.41), attributed to hydrogens 1″ and 1′, ‘‘respectively, as well as the presence of carbonyl hydrogens in the region of δH 3.15–3.71. The ^13^C NMR spectrum (Figure S2) shows a signal at δC 179.607, which is typical of a chelated carbonyl. It shows signals in the region of δC 68.61–78.22, and two signals at δC 102.476 and 100.008, referring to the anomeric carbons 1″ and 1′”, respectively. These results confirm the existence of two sugar units in this flavonoid. For AM1, heteronuclear single-quantum HSQC and HMBC analyses were also performed. In the HSQC spectrum (Figure S3), it is possible to verify the correlation of hydrogens 6 (δH 6.35), 8 (δH 6.60), 3′ (δH 7.05), 6′ (δH 8.08), 1″ (δH 5.22), 6″ (δH 3.83), 1‴ (δH 4.53) and 6‴ (δH 1.12) with carbons 6 (δC 99.23), 8 (δC 93.35), 3′ (δC 117.52), 6′ (δC 132.43), 1″ (δC 102.48), 6″ (δC 68.61), 1‴ (δC 100.01) and 6‴ (δC 17.92). The HMBC spectrum Figure S4) shows the correlation of hydrogen 6 (δH 6.35) with carbons 5 (δC 163.04), 8 (δC 93.35), and 10 (δC 105.70), the correlation of hydrogen 8 (δH 6.60) with carbons 2 (δC 159.40), 6 (δC 99.23), 7 (δC 167.47), and 10 (δC 105.70), the correlation of hydrogen 2′ (δH 124.27) with carbons 2 (δC 159.40), 1′ (δC 123.62), and 4′ (δC 151.83), the correlation of hydrogen 3′ (δH 7.05) with carbons 1′ (δC 123.62), 3′ (δC 135.961), and 4′ (δC 151.83), the correlation of hydrogen 6′ (δH 8.08) with carbons 2 (δC 159.40), 4′ (δC 151.83), and 5′ (δC 117.52), the correlation of the anomeric hydrogen 1″ (δH 5.22) of the glucose unit with carbon 3 (δC 136.05) of the flavonoid, the correlation of the 1‴ anomeric hydrogen (δH 4.53) of the rhaminose unit with the 6″ carbon (δC 68.61) of the glucose unit, and the correlation of the hydrogen 6‴ (δH 1.12) with carbons 4‴ (δC 73.40) and 5‴ (δC 69.77). Both hydrogen and the aforementioned carbons belong to the rhaminose unit. The ^1^H, ^13^C, HSQC, and HMBC NMR spectra are in agreement with the structure shown in Figure 4C. To the best of our knowledge, no flavonoids with this description have been reported in the literature.

For AM2, the ^1^H NMR spectrum shows a doublet at δ 6.23 (*J =* 2.10 Hz), referring to hydrogen 6; a doublet at δH 6.42 (*J =* 2.10 Hz) in the region of aromatic hydrogens, referring to hydrogen 8; a doublet at δH 7.68 (*J =* 1.85 Hz), referring to hydrogen 2′; a doublet in δH 6.89 (*J =* 8.29 Hz), referring to hydrogen 5′; and a double doublet at δH 7.65 (*J =* 8.41 and 2.10 Hz). The spectrum presents a characteristic model of the quercetin derivatives. The ^1^H NMR spectrum also exhibited two simplets (δH 3.90 and 3.97) with an integration value of approximately 3, indicating the presence of two methoxyls in the flavonoid. It is also possible to observe two sugar units through the anomeric hydrogen signals at δH 5.13 (*J =* 7.79 Hz) and δH 4.54 (*J =* 0.74 Hz), which are attributed to the 1″ hydrogens and 1′, respectively, in addition to the presence of carbinolic hydrogens in the region of δH 3.15–3.71. Because of the coupling constant of the doublet absorbing at δH 5.13 (*J =* 7.79 Hz), it is possible to establish the presence of β-D-glucose. In addition to the coupling constant of the doublet absorbing in δH 4.54 (*J =* 0.74 Hz) and the presence of a doublet in δH 1.14 (*J =* 6.31 Hz) integrating for three hydrogens, it is possible to establish the presence of α-L-raminose [21]. The ^13^C NMR spectrum presented a signal at δC 179.48, typical of chelated carbonyl, as well as signals in the region of δC 68.61–78.22 and two signals at δC 104.37 and 102.48, referring to the anomeric carbons 1′′ and 1, respectively, thus confirming the existence of two sugar units in this flavonoid. HSQC and HMBC analyses were also performed. The ^1^H, ^13^C, HSQC, and HMBC NMR spectra were compared with the ^13^C NMR data reported in the literature [22] and were in agreement with the flavonoid ombuin-3-rutinoside, indicating that the AM2 of the AM fraction was ombuin-3-rutinoside (Figure 4D).

## 3. Discussion

The use of medicinal plants in phytotherapy is an ancient and widely practiced tradition in various cultures [1,11]. The current limitations of allopathic medicine in terms of offering definitive relief from this complex disease demand the investigation of medicinal plant extracts with multiple active ingredients [1,2,3]. Phytochemical and pharmacological studies on these compounds have led to the identification of bioactive substances that provide safe and cost-effective therapeutic alternatives. These bioactive substances offer valuable insights into the development of new drugs and treatments under complex conditions [23]. Among these compounds, flavonoids stand out for their specialized biological functions, such as defense against herbivores and protection from UV rays, in addition to their well-documented cardiovascular and vasorelaxant effects [20,24,25].

In the Erythroxylum genus, the presence of flavonoids and tannins has been demonstrated in several species [20]; however, to no, studies have specifically evaluated the potential of *Erythroxylum campestre* in the treatment of hypertension. The present study revealed that the methanolic fraction of *E. campestre* (ECM-ppt-M) is rich in glycosylated flavonoids, including rutin and kaempferol-3-rutinoside. Most importantly, this fraction, with its full complement of compounds, contributes to significant reductions in blood pressure and promotes vasodilation. These effects, which are particularly pronounced in hypertensive animals, underscore the therapeutic potential of *E. campestre* in phytotherapy for managing cardiovascular conditions such as hypertension.

The results demonstrated that ECM-ppt-M treatment induced bradycardia and vasodilation in the renal and aortic territories, leading to a reduction in MAP in hypertensive rats. This antihypertensive effect likely arises from the extract’s ability to simultaneously decrease cardiac output and reduce total peripheral vascular resistance. The unique advantage of using this extract lies in its capacity to target multiple mechanisms at once to lower blood pressure—by reducing both cardiac output and peripheral resistance. In contrast, achieving similar effects with allopathic medicine would require at least two drugs (such as beta-blockers and inhibitors of the renin–angiotensin–aldosterone system), which increases the risk of toxicity. The *E. campestre* extract, therefore, presents a potentially safer, more integrated approach.

In spontaneously hypertensive rats (SHRs), there is an increase in ROS production, leading to increased oxidative stress, which contributes to the pathophysiology of hypertension [26,27]. Several studies have demonstrated that antioxidants effectively reduce MAP in different experimental models of hypertension [28,29]. Moreover, flavonoids can act as reducing and scavenging agents for free radicals [30,31]. Thus, by infusing ECM-ppt-M, it is plausible that the potential antioxidant action of this fraction reduced vascular oxidative stress, thereby causing vascular relaxation and a consequent reduction in vascular resistance and MAP.

The phytochemical characterization of extracts often provides a basis for associating their actions with active principles. Indeed, phytochemical analyses have shown that the presence of flavonoids in the extracts of phytotherapeutic plants may be related to antihypertensive and vasorelaxant activities [32]. Thus, once the antihypertensive and vasodilatory effects of ECM-ppt-M were determined and the potential antihypertensive action of flavonoids in phytotherapy was considered, the present study evaluated the chemical structure composition of ECM-ppt-M using NMR.

NMR analysis revealed that the E1F fraction was a characteristic model of quercetin derivatives. Based on the coupling constant of the doublet absorbing at δH 5.00 (*J =* 7.93 Hz), the presence of β-D-glucose can be established. Similarly, based on the coupling constant of the doublet absorbing at δH 4.42 (*J =* 1.21 Hz) and the presence of a doublet at δH 1.02 (*J =* 6.19 Hz), performing integration for three hydrogens, the presence of α-L-rhamnose can be established [33]. The ^1^H, ^13^C, HSQC, and HMBC NMR spectra and the comparison with ^13^C NMR data reported in the literature [34] were consistent with the identification of the flavonoid rutin as quercetin-3-rutinoside in the EF1 fraction. The F1 fraction, presenting a characteristic model of kaempferol derivatives, had a coupling constant value of hydrogen 1″ at approximately *J =* 7.16 Hz, suggesting that the sugar is linked to the flavonoid by the β-face. Therefore, based on the coupling constant of the doublet at δH 4.53 (*J =* 1.01 Hz) and the presence of a doublet at δH 1.14 (*J =* 6.17 Hz) performing integration for three hydrogens, the presence of α-L-rhamnose can be established [33]. Additionally, when comparing the ^1^H HSQC and HMBC spectra with ^13^C NMR data reported in the literature [35], the results were consistent with the identification of the flavonoid kaempferol as kaempferol-3-rutinoside. The AM1 fraction appears to be a characteristic model of quercetin derivatives, with the possible presence of β-D-glucose based on the coupling constant of the doublet absorbing at δH 4.53 (*J =* 1.41 Hz) and the presence of a doublet at δH 1.12 (*J =* 5.96 Hz), performing integration for three hydrogens, indicating the possible presence of α-L-rhamnose [33]. Moreover, our data confirmed the existence of two sugar units in this flavonoid, with both hydrogen and the aforementioned carbons belonging to the rhamnose unit. Thus, this chemical configuration suggested the characterization of a novel flavonoid.

In summary, we have demonstrated the unprecedented therapeutic potential of *Erythroxylum campestre* by combining advanced phytochemical techniques with hypertensive animal models to identify active compounds and assess their biological activities. This study provides several novel contributions, including the assessment of a glycosylated flavonoid-rich leaf extract from *E. campestre* (ECM-ppt-M). We identified, isolated, and characterized key glycosylated flavonoids—such as rutin, kaempferol-3-rutinoside, and ombuin-3-rutinoside—using HPLC, LC-MS, and NMR. Importantly, the extract exhibited pronounced hypotensive and vasodilatory effects, particularly in hypertensive models, by simultaneously reducing both cardiac output and total peripheral vascular resistance. This dual action underscores the potential of *E. campestre* as a holistic therapeutic approach for managing cardiovascular diseases. These findings advance the bioprospection of phytotherapy for cardiovascular diseases, as the glycosylated flavonoids in the extract enhance solubility, bioavailability, and stability in the body. As noted by Yonekura-Sakakibara et al. (2014), the glycosylation of flavonoids is essential for their stable accumulation [36], potentially amplifying their health benefits [37].

Altogether, our findings support the exploration of glycosylated flavonoids for use in pharmacokinetic evaluations, thereby expanding the scope of cardiovascular disease research and treatment. Future studies, including preclinical and clinical trials on ECM-ppt-M, could position this extract as a promising therapeutic alternative to synthetic drugs, potentially reducing the risk of toxicity and adverse effects.

## 4. Methods

### 4.1. Acquisition and Characterization of E. campestre Extract

#### 4.1.1. Extraction Process

The collected leaves (2.3 kg) were dried in a circulating air oven at 45 °C, resulting in 805.87 g of dehydrated material. The dried material was ground using a Willey knife mill and macerated using 96% methanol. The obtained liquid was concentrated using a rotary evaporator, resulting in a viscous liquid, referred to as ECM, with a mass of 199.44 g. This extract was then solubilized in a mixture of methanol and water (1:3) to ensure the complete solubilization of all constituents. The methanolic extract was further fractionated through liquid–liquid partitioning. The precipitate from the methanolic aqueous solution was fractionated by open-column chromatography (ϕ = 3 cm × h = 20 cm), with microcrystalline cellulose D (20–160 µm) acting as the stationary phase, and eluents were used in the order of increasing polarity (dichloromethane, ethyl acetate, and methanol). This process yielded the following fractions: dichloromethane (ECM-D), ethyl acetate (ECM-A), and methanol (ECM-M). These were obtained in masses of 1.35 g, 4.36 g, and 11.03 g. The plant material samples, as well as extracts, fractions, and isolated compounds, were protected from light and heat because of concerns about the oxidation of the compounds. To achieve this, they were refrigerated at 5 °C and stored in amber bottles, and the analyses were carried out in a light-controlled environment [38,39].

#### 4.1.2. Isolation of the Organic Fraction by Chromatographic Techniques

The ECM-M sample (11.03 g) was fractionated in a classic preparative column (h = 14 cm and ϕ = 1.5 cm) using Sephadex LH20 (18–111 μm dry) as the stationary phase and methanol as the mobile phase. After elution, 81 vials with a capacity of 10 mL were collected. Following Thin-Layer Chromatography (TLC), the collected substances were grouped based on their retention factors (Rf), resulting in five fractions. Among these, the third fraction (vials 40–43) underwent further fractionation in a preparative column (h = 43.2 cm and ϕ = 2.0 cm) using Sephadex as the stationary phase and methanol as the mobile phase. This procedure yielded 151 vials with a capacity of 10 mL. The substances collected were grouped according to their Rf values from TLC assays, resulting in 13 fractions. The first five fractions (I, II, III, IV, and V) were discarded because of the different Rf values and low masses observed by TLC. The remaining eight fractions (VI, VII, VIII, IX, X, XI, XII, and XIII) were subjected to qualitative analysis by high-performance liquid chromatography and quantitative analysis using reversed-phase (HPLC/RP) chromotography.

#### 4.1.3. Qualitative Analysis

To analyze the eight selected fractions (VI, VII, VIII, IX, X, XI, XII, and XIII), approximately 0.02 mg of each vial was separated by HPLC using a Luna analytical column (C18, 250 mm × 4.6 mm, 5 μM). The eluents consisted of solutions A (methanol) and B (phosphoric acid Solution A pH 2.62) at a flow rate of 1.0 mL/min. The gradient elution varied from 100 to 0% of A for 8 min, from 0 to 50% of A for 8 min, and from 50 to 100% of A for 3 min, and then stabilized at 100% of A for another 1 min, totaling 20 min. Detection was performed by UV-Vis at 360 nm.

#### 4.1.4. Quantitative Analysis

After confirmation that samples were equal by HPLC analysis, the eight fractions were combined, forming a mass of 684.7 mg named ECM-ppt-M. This was analyzed by HPLC using a Luna semi-preparative column (C18, 250 mm × 10.0 mm, 5 μM). The mobile phases were solution A (methanol) and solution B (phosphoric acid solution, pH 2.62), with a flow rate of 1.0 mL/min. The gradient elution varied from 50 to 80% A for 35 min, from 80 to 95% A for another 5 min, and from 95 to 50% A for more than 2 min, and then stabilized at 50% A for another 3 min, totaling 45 min of analysis. Detection was performed at a wavelength of 360 nm.

#### 4.1.5. Mass Spectrometry (MS)

Mass spectrometry (MS) was performed on a Bruker micrOTOF-Q III instrument. Electrospray ionization (ESI) was performed in negative and positive modes. Maintained at 0.4 bar, high-purity nitrogen (>98%) was used as the nebulizer gas, and the capillary voltage was set to 4500 V. The conditions of the quadrupole time-of-flight (Q-TOF) mass analyzer were optimized to perform the analysis. Mass spectra were acquired and processed using the Bruker Compass Data Analysis software v.4.3.

#### 4.1.6. Analysis by Nuclear Magnetic Resonance Spectroscopy (NMR)

NMR spectra were acquired using a Bruker Avance III instrument (11.75 T). A 5 mm broadband probe head with a z-gradient was used at 25 °C. Operating frequencies were 500.13 MHz for ^1^H and 125.75 MHz for ^13^C. Heteronuclear single quantum coherence (HSQC) and heteronuclear multiple-bond coherence (HMBC) experiments are occasionally conducted. Deuterated methanol (CD_3_OD) was used as the solvent and tetramethylsilane (Si(CH_3_)_4_, TMS) was used as the internal standard. Experiments were conducted in 5 mm diameter tubes, and data were collected using Topspin 3.5 software developed by Bruker Biospin.

### 4.2. In Vivo Experiments

#### 4.2.1. Animals

Adult male Wistar (normotensive) and spontaneously hypertensive rats (SHRs) (10–12 weeks, 250–350 g), obtained from the central animal facility of the Federal University of Goias, were used. These animals were housed at the Department of Physiological Sciences in polypropylene cages (45 cm × 30 cm × 15 cm) under controlled conditions of a 12 h light–dark cycle (07:00 a.m. to 07:00 p.m.) and a temperature of 22.0 ± 2 °C. All experimental procedures followed the Guidelines for the Care and Use of Laboratory Animals approved by the Ethics Committee of the Federal University of Goias (protocol number 064/17).

#### 4.2.2. Surgical Procedures

The animals were anesthetized using isoflurane (isoflurane 2%, Tanohalo, Cristália, Itapira, SP, Brazil) in 100% O_2_ prior to the catheterization of the femoral artery and vein. After vein catheterization, anesthesia was maintained by the administration of urethane (400 mg·kg^−1^, i.v., Sigma-Aldrich, St. Louis, MO, USA). Tracheostomy was performed to reduce airway resistance. The renal blood flow (RBF) and aortic blood flow (ABF) baselines were recorded using a miniature probe placed around the left renal artery and abdominal aorta. The body temperature of the rats was maintained between 36 and 37 °C on a heating pad throughout the experimental procedures. Following the stabilization of cardiovascular parameters, ECM-ppt-M (1, 2, 3, and 6 mg∙kg^−1^) and vehicle (5% DMSO in distilled water) were randomly administered to normotensive and hypertensive rats.

#### 4.2.3. Recording of Cardiovascular Parameters

Pulsatile arterial pressure (PAP) was recorded continuously through the arterial cannula, which was connected to an amplifier-coupled pressure transducer (Bridge Amp FE221; ADInstruments, Colorado Springs, CO, USA). The data were digitized at a frequency of 1000 samples/s using an analog-to-digital converter (PowerLab 4/25, ML845, ADInstruments, Colorado Springs, CO, USA). The mean arterial pressure (MAP) was calculated from the integral of the PAP signal using LabChart software (v.7.3.7, ADInstruments, Colorado Springs, CO, USA). The heart rate (HR) was calculated as the instantaneous frequency from the PAP signal (PowerLab 4/25, ML845, ADInstruments, Colorado Springs, CO, USA).

The miniature probes were connected to a T206 flow meter (Transonic Systems, Inc., Ithaca, NY, USA) to record RBF and ABF. The signals obtained were recorded using an acquisition and data analysis MP150 system (PowerLab 4/25, ML 845; AD Instruments, Bella Vista, Australia). The data were digitized at a sampling frequency of 200 samples/s. Changes in the RBF and ABF were calculated as percentages relative to the baseline (%RBF and %ABF). Renal vascular resistance (RVR) and aortic vascular resistance (AVR) were determined using MAP/RBF and MAP/ABF ratios, respectively. Variations in RVR and AVR were expressed as percentage changes in baseline values (%RVR and %AVR).

#### 4.2.4. Statistical Analysis

Statistical analyses of cardiovascular parameters were performed using GraphPad Prism software (v. 9.01). Cardiovascular parameters are expressed as mean ± standard error of the mean (SEM). MAP, HR, RBF, ABF, RVR, and AVR variations were analyzed using two-way analysis of variance (ANOVA), followed by Tukey’s post hoc test.

## 5. Conclusions

The results obtained in this study demonstrated for the first time that ECM-ppt-M from *E. campestre* exhibits antihypertensive and vasodilatory effects in hypertensive animals. Furthermore, the HPLC-DAD method, which efficiently separated flavonoids from the ECM-ppt-M fraction, facilitated the identification of quercetin-3-rutinoside (rutin), kaempferol-3-rutinoside, ombuin-3-rutoside, and a novel flavonoid. As demonstrated in this study, the biological effects of ECM-ppt-M will further spur the evaluation of the extract and its isolated compounds for the treatment or prevention of cardiovascular diseases. These properties make this species and its derivatives a significant bio-input for the production of medicines for human and veterinary purposes.

## Figures and Tables

**Table 1:** Fractions of the crude methanolic extract (ECM).

ECM Mass	Solvent	Mass Obtained (g)	% in Relation ECM	Code
	Hexane	5.28	2.65	ECM-H
	Dichloromethane	0.80	0.40	ECM-D
199.44 g	Ethyl acetate	18.70	9.38	ECM-A
	Precipitate	58.73	29.45	ECM-ppt
	Residue	115.93	58.12	-

**Table 2:** Fractionation of precipitated material (ECM-ppt).

ECM-ppt Mass	Solvent	Mass Obtained (g)	% in Relation ECFM	Code
	Dichloromethane	1.35	2.30	ECM-ppt-D
	Ethyl acetate	4.36	7.42	ECM-ppt-A
58.73 g	Methanol	11.03	18.78	ECM-ppt-M
	Residue	41.99	71.50	-

**Table 3:** Mass spectrometry data of fractions E1F, F1, and AM.

Fraction	Molecular Ion *m/z*	Base Peak *m/z*	Ionic Fragments *m/z*
E1F	1245.3102	634.1579	815.5211/771.4944/727.4685/ 551.3627/463.3088/303.0535/ 245.0883/139.0523
F1	1198.7095	595.3916	1122.7155/1024.7452/926.7677/ 828.7928/683.4451/639.4170
AM	1272.3026	633.1480	903.5696/815.5190/727.4661/ 547.4085/501.3795/419.2803/ 303.0523/229.0937/175.0460/ 127.0252

## Data Availability

The data presented in this study are available on request from the corresponding author due to legal and privacy reasons.

## Supplementary Materials

The supporting information can be downloaded at: https://www.mdpi.com/article/10.3390/ph17111456/s1.
