# Supplementary material for: Cardiovascular Effects of a Glycosylated Flavonoids-Rich Leaf Extract from Brazilian Erythroxylum campestre: A Potential Health Bio-Input
Source: Pharmaceuticals (Basel). 2024 Oct 30;17(11):1456. doi: 10.3390/ph17111456 (PMC11597499; doi:10.3390/ph17111456)

Supplementary material

Table S1. ^{1}H (MeOD, 500 MHz) and ^{13}C (MeOD, 125 MHz) NMR data of the AM fraction with values assigned to compound AM1 compared to literature values ^{1}H (DMSO-d_6, 500 MHz) and ^{13}C (DMSO-d_6, 125 MHz).

Position	δH (ppm), *J* (Hz)		δC (ppm)	
	**AM1**	**Literature**	**AM1**	**Literature**
2			**159.397**	156.5
3			**136.054**	133.2
4			**179.607**	177.3
5			**163.038**	161.2
6	**6.35 (*d, J = 2.13*)**	6.20 (*d, J = 2.1*)	**99.231**	98.7
7			**167.474**	164.5
8	**6.60 (*d, J = 1.86*)**	6.40 (*d, J = 2.1*)	**93.346**	93.6
9			**158.491**	156.4
10			**105.704**	103.7
1’			**123.619**	121.5
2’		(7.53 (*d, J = 2.0*)	**124.274**	116.2
3’			**135.691**	115.2
4’			**151.829**	148.5
5’	**7.05 (*d, J = 8.73*)**	6.83 (*d, J = 9.0*)	**117.517**	115.2
6’	**8.08 (*d, J = 9.07*)**	7.75 (*dd, J = 7.8 e 2.4*)	**132.429**	121.0
1’’	**5.22 (*d, J = 7.88*)**	5.44 (*d, J = 7.2*)	**102.476**	101.2
2’’			**77.295**	75.8
3’’			**75.785**	74.0
4’’			**71.539**	69.9
5’’			**78.210**	76.4
6’’	**3.83 (*d, J = 11.06*)**		**68.614**	66.9
1’’’	**4.53 (*d, J = 1.41*)**	4,38 (*d, J = 1.0*)	**100.008**	100.7
2’’’			**72.307**	70.5
3’’’			**72.160**	70.3
4’’’			**73.999**	71.8

5'''			**69.770**	68.2
6'''	**1.12 (*d, J = 5.96*)**	0,99 (*d, J = 6.0*)	**17.924**	17.6

"$\boldsymbol{\delta_H}$" – Hydrogen chemical shift; "$\boldsymbol{\delta_C}$" – Carbon chemical shift; "*J*" – Coupling constant; "d" – Doublet; "dd" – Double doublet; "Hz" – Hertz; "Literature" – Moura, Vilega, and Santos (2011); "ppm" – Parts per million.

Figure S1. ^{1}H NMR spectrum (MeOD, 500 MHz) of the AM fraction, highlighting the AM1.

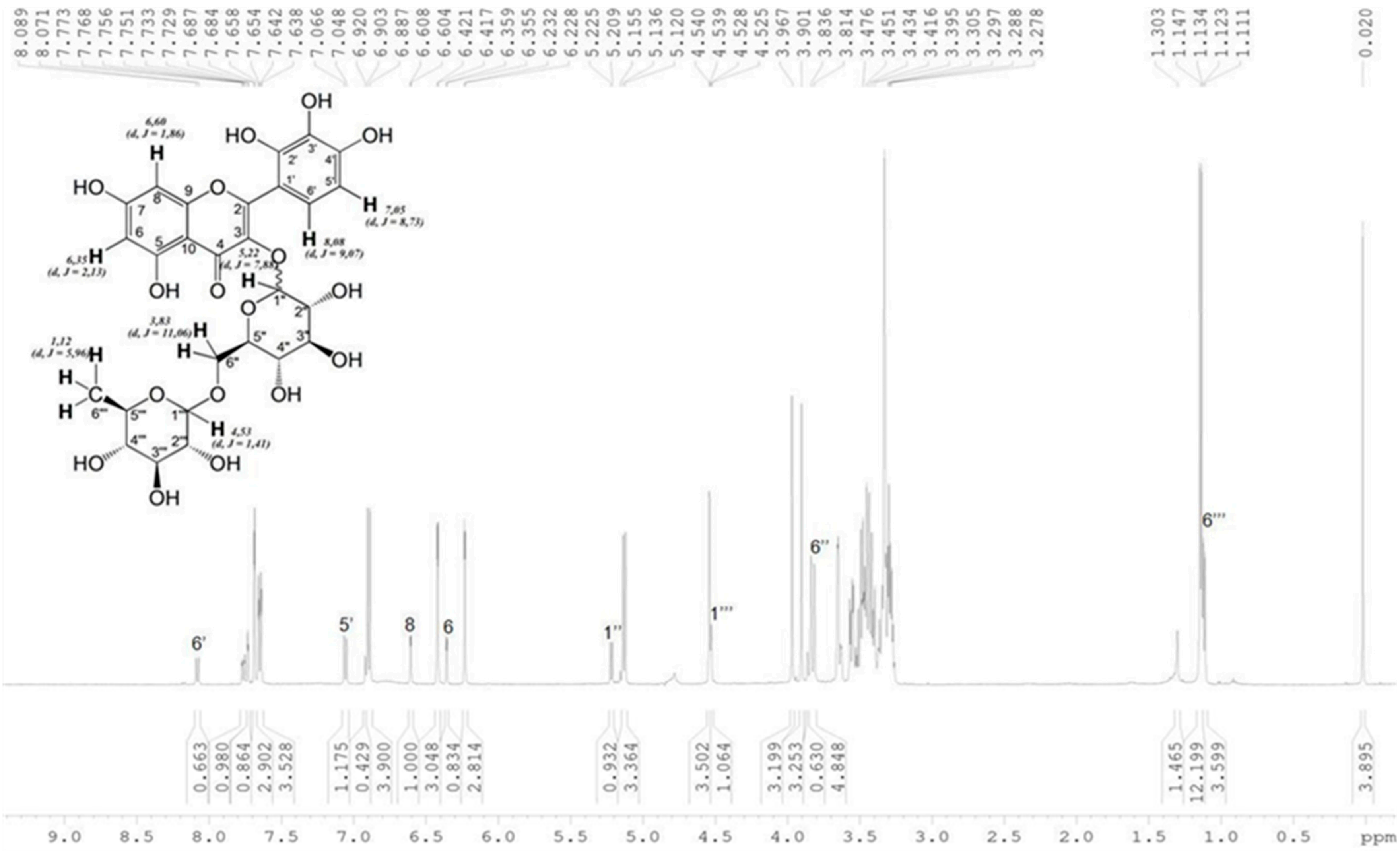

Figure S2. ^{13}C NMR spectrum (MeOD, 125 MHz) of AM fraction, highlighting AM1.

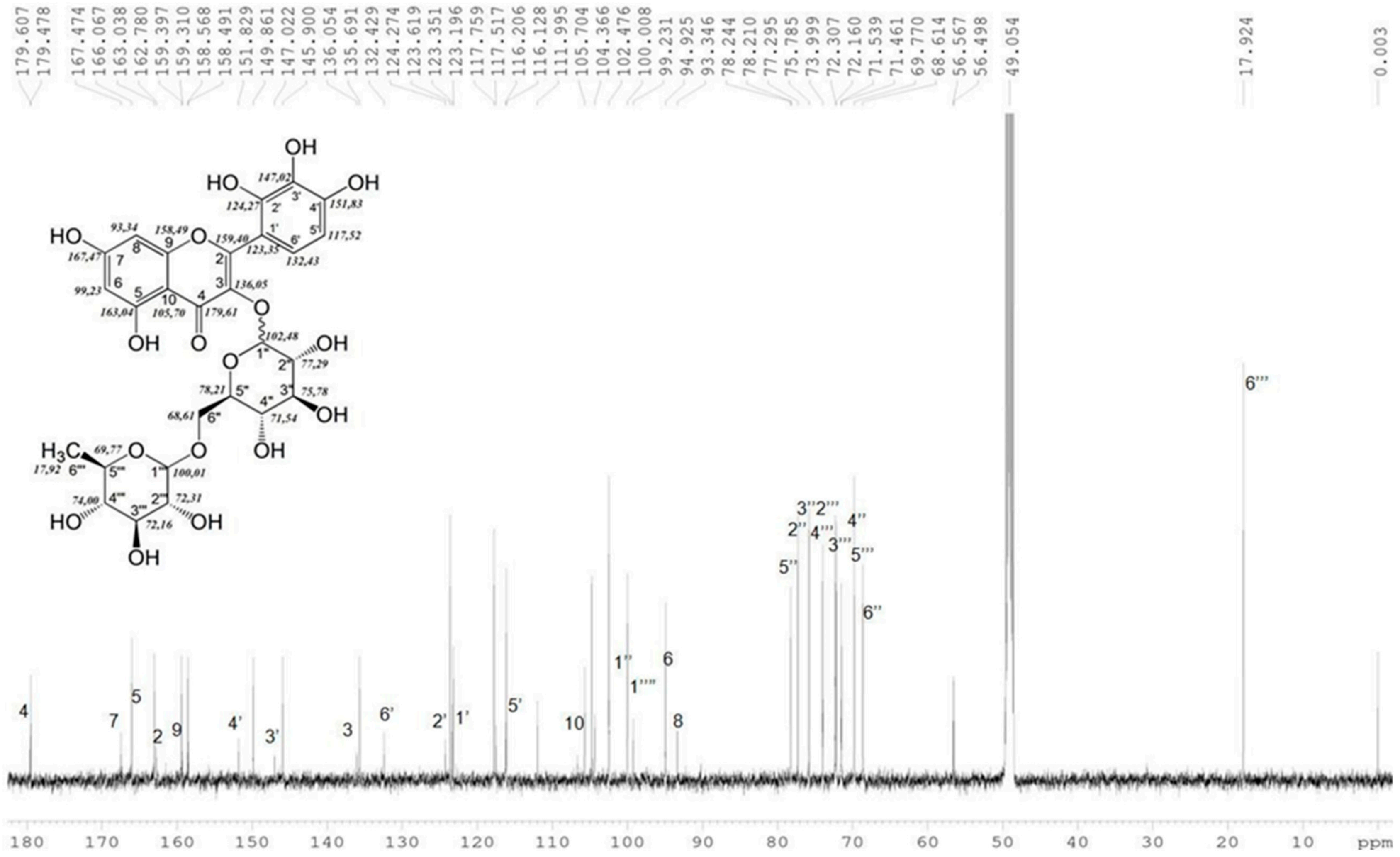

Figure S3. HSQC Contour Map of the AM fraction (MeOD, ^{1}H 500 MHz; ^{13}C 125 MHz), highlighting the AM1.

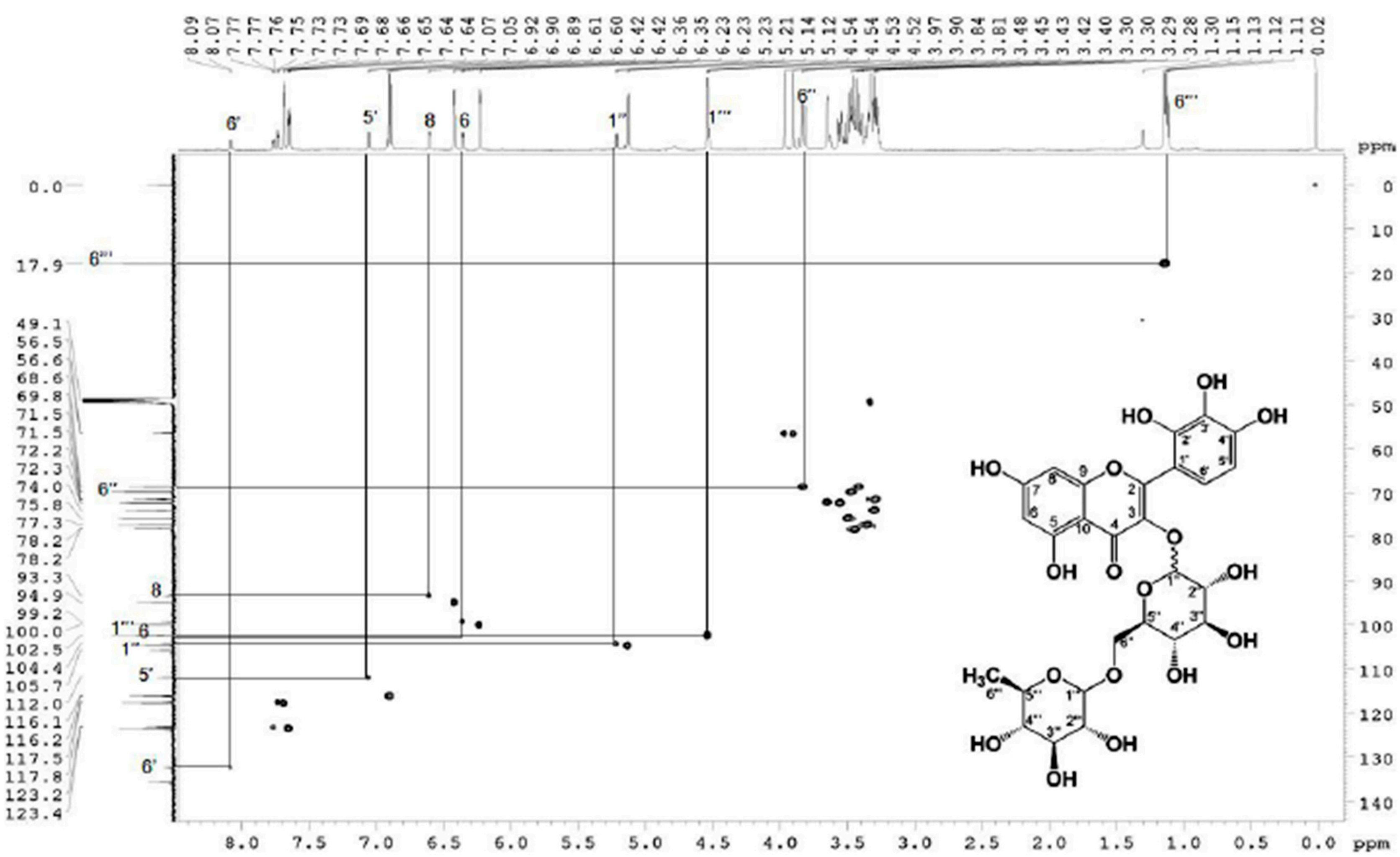

Figure S4. HMBC Contour Map of the AM fraction (MeOD, ^{1}H 500 MHz; ^{13}C 125 MHz), highlighting the AM1.

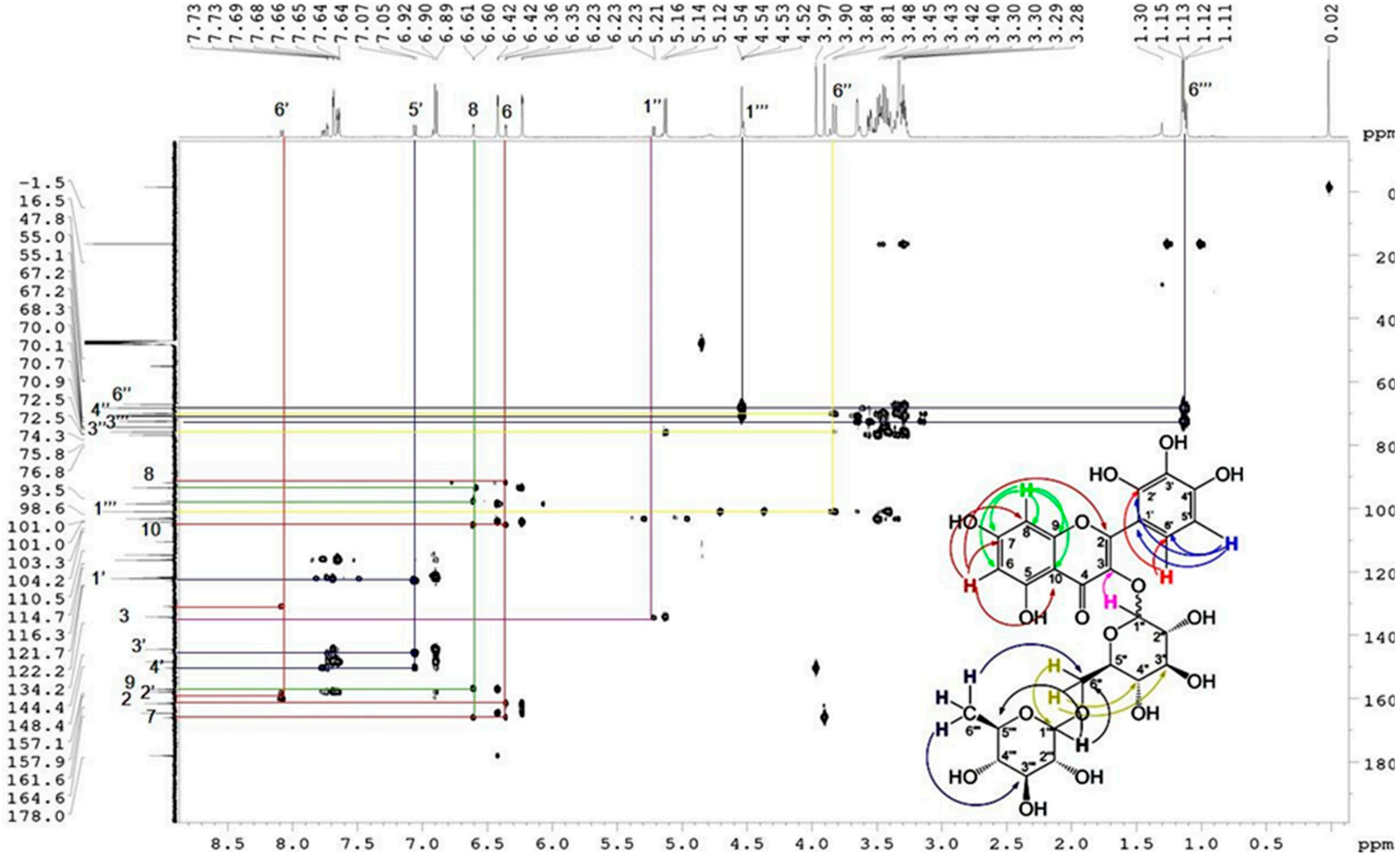

Supplement: Supplementary file 1 [file pharmaceuticals-17-01456-s001.zip › pharmaceuticals-3243146-supplementary.pdf]
